# Notes and News

**Published:** 1887-04-16

**Authors:** 


### NOTES AND NEWS.

COLLECTED AND COMMUNICATED.

SIR WILLIAM JENNER, M.D., has, for the seventh time, been elected President of the Royal College of Physicians.

DR. E. SYMES THOMPSON has arranged to give a series of free lectures at Gresham College, Basinghall Street, on "The Blood." The dates fixed are April 19, 20, 21, and 22.

DR. T. STRETCH DOWSE has been appointed one of the Physicians of the Heart Hospital, Soho Square.

ON Friday, the 1st inst., Lady Brabazon and the members of the Kyrle Society gave a musical entertainment for the patients of the Heart Hospital, Soho Square.

MR. HENRY F. SEMPLE, M.R.C.S., L.R.C.P., has been elected to fill the vacancy in the office of house-surgeon to the Bristol Hospital for Sick Children, caused by the resignation of Mr. E. Leonard Lees, M.B., C.M., M.R.C.S.

THE Honorary Secretary of the Mold Cottage Hospital, in thanking us for the notice of his institution which recently appeared in our columns, says, "I have taken your publication from the commencement; our nurse is very pleased with it, *romances* and all." Thus it would seem that our lady-readers, in Wales at any rate, are not quite so (may we venture to say?) hypercritical as those nearer home.

MR. H. THORNHILL ROXBY, the Secretary of the After-Care Association for Poor and Friendless Female Convalescents, writes from Emblewood, Osbaldeston Road, N., to inform us that the committee of the Association are most anxious to obtain the loan of a suitable house to be used as a convalescent home for the poor women who are the objects of the Society's care. Funds are also greatly needed to carry on the work of this Association, which has the Archbishop of Canterbury and Cardinal Manning for Vice-Presidents.

IT has been unanimously resolved to earnestly invite the various trade, friendly, temperance, and benefit societies of East and North-Eastern London to cooperate for the purpose of securing the success of a Jubilee "Banner Service" at the Pavilion Theatre, in aid of the Morley House Working Men's Convalescent Home, St. Margaret's, near Dover. The theatre has been gratuitously placed at the disposal of the promoters of this service.

WE are informed that the Council of the Hospital Saturday Fund have issued collection sheets to all the principal industrial establishments of London, and they again appeal to employers of labour, foremen, and others, so that the collection during the present year of Jubilee may show a large increase upon previous years. All metropolitan dispensaries and other medical charities are to be invited to participate in the fund for 1887.

ON Thursday week the last of this season's entertainments to the patients of the Cancer Hospital, Brompton, took place in the out-patients' waiting-room, when a programme of much interest was ably carried out. Most of the officials and a number of visitors attended. The patients appeared highly delighted with the evening's entertainment. The chaplain (the Rev. N. Liberty) proposed a hearty vote of thanks to the *artistes* for their assistance. Mr. Hughes (the secretary), in acknowledging the compliment, said that the ladies and gentlemen who had helped them that evening would be very pleased at any time to come down and relieve the monotony of hospital life by a few songs, and so divert the attention of the sufferers from their dreadful disease.

ON the 31st ult. Sir E. Sieveking, M.D., presided at an important meeting of physicians and surgeons, held at the offices of the Order of St. John, St. John's Gate, Clerkenwell, the object of which was to consider the best means of promoting that branch of the work of the St. John's Ambulance Association connected with the conveyance of the sick from their homes, or from the scene of accident to the hospital. Mr. Furley described horse ambulance carriages, litters, stretchers, and other ingenious contrivances which he has devised. Improved facilities for the conveyance of the sick to hospitals are one of the most pressing needs of the day, and we are pleased to see that the Association, which has done such good work in rendering first aid to the wounded, is turning its attention to this question.

WE have received the following definition of a hospital by a grumbler, whose views will no doubt amuse, as well as enlighten the reader:—A place where one sadly lacks the comfort of home. A public bedroom. A place where food and medicine is given to you at stated intervals whether you like it or not. A place where, no matter how racked with pain you are, you must lie still, or you will surely get into "nurse's black" books for keeping your bed untidy."

THE following vacancies are announced this week, the amount of the salary being placed in each case after the name of the institution :—

**Dispenser.**

St. Pancras Infirmary, Highgate, N.W.—£100.

**Trained Nurses.**

Cottage Hospital, Blackheath. Nurse and Housekeeper.
Establishment for Invalid Gentlewomen, 90, Harley Street. Hospital, One.—£25.
Nurses' Institute, Canterbury. Four.
Parish of All Saints', Scarborough. One.

**Probationers.**

Lowestoft Hospital. One.
Nurses' Institute, Canterbury. One.
Victoria Hospital, Burnley. One.—£9.

---

THE Hospital Saturday Fund will, it is hoped, be materially assisted this year by the cabmen of London. Last Friday, Sir Edmund Henderson, in presiding over a meeting at the Shaftesbury Memorial Hall, Great Ormond Street, called in furtherance of this object, remarked that of all charities of the metropolis, he knew of none which were more deserving of support than our hospitals. In 1885, on the occasion of the first special cab trade collection, £169 was raised, but last year the amount fell to £141. When we remember that the men employed in the cab trade of the metropolis are to be numbered by tens of thousands, it is obvious that the aggregate contribution ought to be very much greater. The calling of the London cabman is one which is open to considerable risk of accident, and it is therefore to be hoped that the recognition of this fact will lead to a much more general response among cabdrivers when the next collection is made.

---

To whom is the origin of Hospital Sunday due? Mr. H. C. Burdett, in a pamphlet on the origin, progress, and development of Hospital Sunday and Hospital Saturday, has gone very carefully into the disputed claim. He points out that, while the late Dr. Wakley may be correctly described as the founder of Hospital Sunday in the metropolis, the movement first originated in Birmingham ; and he shows that, while the suggestion of an annual collection in all places of worship originated with the great philanthropist, the late Thomas Barber Wright, the foundation of Hospital Sunday was in reality due to the Rev. Canon Miller. But yet another aspirant to the honour has been brought forward. Mr. Frank Davidson, of West Bromwich, writing to the *Birmingham Post*, denies the claim of Mr. T. B. Wright, remarking, "The idea originated with my late father, Alfred Davidson, who at the time was collecting materials for a history of St. Martin's Church, and was upon friendly terms with the late Rector. Canon Miller's reply to my father's suggestion was, 'Well, Mr. Davidson, I'll preach a sermon if you will write an article.'" He adds, "So long as the credit of originating the movement lay with Dr. Miller, who was, undoubtedly, the most influential mover in the matter, I was unwilling to claim the authorship upon my late father's behalf, but I cannot allow it to be ascribed to a man who really had little more to do with the affair than to permit an article to appear in the *Midland Counties Herald*."

THE difficulties which have been experienced during the past year with respect to the medical staff of the Essex and Colchester General Hospital, are fully set forth in the sixty-seventh annual report of the institution, which has lately reached us. Dr. Veale and Dr. Bawtree have both retired, the one in March and the other in August 1886 ; and in spite of great efforts on the part of the committee, it has not yet been found possible to obtain the services of other physicians, practising purely as such. The work of the hospital has, however, been carried on by the house-surgeons, and 531 patients were admitted, the average residence of each being forty-one days and a half. The accounts show a deficit of £14 on the year, but this is only apparent, not real, as over £800 of the total £3,441 has been expended in the purchase of three per cent. consols ; and the committee are therefore able to speak of the financial condition of the hospital as prosperous and satisfactory.

---

THE annual meeting of the supporters of St. John's Hospital, Twickenham, was held on the 12th February, the vice-president, Mr. G. G. Mackintosh, being in the chair. There has been a most marked and satisfactory progress in the condition of the hospital during the last twelve months, and the ten beds which it contains have been continually occupied. Of the 127 cases under treatment, 86 were cured and 23 relieved ; whilst five deaths only took place. The clergy and ministers in the district have agreed to observe Hospital Sunday on one day in the year, so that collections for the hospital may be made simultaneously in every church and chapel in the parish. From these sources the sum of £105 was obtained last year, and we cannot help expressing a wish that ministers of all denominations throughout the country would manifest a similar agreement on this important subject. The total receipts amounted to £882 and the expenses to £705, there being thus a balance in hand of £77 for the credit of the current account, a result that cannot fail to be highly satisfactory to the careful managers of the institution.

---

THE Governors and Building Committee of the New Bath Hospital and Convalescent Home at Harrogate, which is being erected on the site of the old buildings, are making strenuous efforts to increase the subscriptions to the Building Fund, so that if possible the new hospital may be opened free of debt. More than fifteen thousand pounds has been promised, but it is stated that a large sum is still required. The charity is an ancient one, for although no hospital was built until 1824, it is recorded that for forty or fifty years before, a fund averaging about £150 annually had been raised by subscription amongst the visitors to Harrogate, and distributed to the poor who resorted there for the benefit of the waters. During 1886, 664 cases were treated ; 94 patients having stayed for a second term of three weeks. There were 432 cases of rheumatism, and many even of old standing were cured, while the majority were much improved in general vigour, and relieved of stiffness, pain, and swelling in their joints. There were also 188 cases of skin disease, and the cures are stated to have been numerous and striking. No deaths occurred during the period.

THE following sums have been recently received by the various Medical Charities, the name of the donor or the source from which they were obtained being given in each instance after the name of the Institution :—

**Donations.**

British Home for Incurables. Miss Ellen Sharpus, 50 guineas.
Charing Cross Hospital. Mr. Frederick Weldon, 20 guineas. Mr. A. Godfree, 10 guineas. Mr. B. F. Barton, 25 guineas.
Chelsea Hospital for Women. Fulham Road. Hon. A. G. Tollemache, £50. Mr. J. A. Whitaker, 10 guineas. Lieut.-General Sir Roger Palmer, Bart., £10.
City of London Lying-in Hospital, City Road. Clothworkers' Company, £25. J. T. Allen, Esq., £10 10s.
Dental Hospital of London. Mr. J. Howard Mummery, 10 guineas. Miss Cohen, 10 guineas.
North-Eastern Hospital for Children, Hackney Road. Mr. Joseph Gurney Barclay, £200.
North London Hospital, Gower Street. Sir R. Palmer, £10. L. F., 10 guineas. Hon. A. G. Tollemache, £100. A. Z., £10.
Royal Free Hospital, Gray's Inn Road. Hon. A. G. Tollemache, £100.
The Mary Wardell Convalescent Home for Scarlet Fever, Stanmore. Company of Leathersellers, 10 guineas.

**Subscriptions.**

Dental Hospital of London. Messrs Claudius Ash and Son, £10.

**Legacies.**

Great Northern Central Hospital. Mr. G. W. P. Bentinck, £1,000.

---

A VERY successful concert was given in the Rink Hall, Blackheath, on Tuesday evening, 29th ult., in aid of the funds of the Miller Hospital, Greenwich. The concert was originated and most ably carried through by the kind efforts of the Senior Surgeon to the Hospital, Mr. Thomas Moore, F.R.C.S., of Lee Terrace, and his musical family, with other friends, members of the Blackheath Conservatoire of Music. Miss Moore, who is a great favourite in the neighbourhood, and an accomplished vocalist, gave three songs with almost more than her usual rich freshness of voice and pathos. Upon her first appearance she was presented with three magnificent bouquets of hot-house and Easter lilies, and her singing was highly appreciated, and won warm and sustained applause. Miss Julia Mark sang several Italian operatic airs with taste, and Miss Cardew showed great talent, and gave much pleasure by her violin solos. Miss Gordon Scott, as well as several gentlemen of acknowledged musical talent, helped to make the concert a success. The large hall was dressed with hot-house flowers, and filled with a fashionable and appreciative audience. The proceeds of the concert amounted to about £100. Much praise is due to Mr. Moore and his family, and they are to be congratulated upon a genuine and deserved success.

---

MR. H. J. MOXON, L.D.S., Dentist to the North Surrey District Schools, Anerley, at the request of the managers gave a popular lecture on the teeth to nine hundred of the senior children and their officers at the schools on the 12th ult. He explained, in the course of his remarks, the advantages of preventive rather than reparative treatment, impressed upon his hearers the necessity of keeping the teeth clean, and dwelt upon the importance of the thorough insalivation of food. The youthful audience were amused by anecdotes, accompanied by illustrations on the black-board. Altogether this, the first address of the kind in Government schools, proved to be a success, and we hope the experiment may be repeated elsewhere.

CAPTAIN WIGGINS, F.R.G.S., recently gave a very interesting and entertaining lecture at Hampstead, in aid of the Hampstead Home Hospital and Nursing Institute. The subject was the lecturer's travels in Arctic Siberia, and a most entertaining and graphic account of a journey, 12,000 miles in length, on dog sledges was given. Captain Wiggins spoke highly of the comfort and luxury to be obtained in a country which has certainly heretofore been regarded rather as a type of misery and privation than anything else, and gave the audience some idea of its scenery by means of dissolving views. Living representatives of Laplanders, Samoyedes, Dolgans, and others tribes, in their native dress, also appeared on the platform, and excited no little interest. A collection in aid of the hospital was made after the lecture, when the sum of £5 15s. was obtained.

---

THE committee of the Silloth Convalescent Institution have recently presented their report for the year 1886 to a meeting of the subscribers, held at Carlisle Town Hall. The number of patients during 1886 was 487, of whom the majority came from Carlisle and Cumberland, although some were received from Westmoreland, Northumberland, Lancashire, and even Scotland. The results of residence in the Institution show that 397 patients gained in weight, whilst the weight of fifty-five others diminished, and in thirty-five other cases the time of stay was either too short, or did not affect the patient appreciably. The finances are in a satisfactory condition, there being a balance of £193 in the bank after payment of all expenses, whilst the capital has by recent legacies been brought up to a total of £5,402. The Institution is deservedly popular in the neighbourhood, and we trust its prosperity and power of doing good and useful work may long continue to increase with years.

---

THE growth of opinion in matters of hospital construction is well exemplified by the medical report of St. Mary's Hospital, Manchester, which was read by Dr. Lloyd Roberts at the annual meeting of the Governors:—"The present institution was erected at a time when the hygienic requirements of a hospital were but imperfectly understood, and although all that is possible has from time to time been done to remove the defects, and improve sanitary conditions, it is obvious that nothing short of a new hospital, constructed in accordance with modern views, will enable those life-saving operations which are the triumph of surgical art to be carried out with the minimum amount of risk to the patient." We hope that a fund for a new hospital may soon be forthcoming, as seems likely from the result of a private canvass, which is eloquent of the liberality of Manchester people. Mr. Oliver Heywood, Mr. James Jardine, Mr. W. H. Houldsworth, M.P., and Mr. F. W. Crossley, with the members of the Management and Medical Committee, each give £1,000 ; there are five donations of £500 each, one of £300, five of £250, and six of £100. Here we have nearly £10,000 subscribed by a few friends of one small provincial hospital with fifty beds, without hesitation or delay. Would that equal generosity were to be found in connection with every medical charity throughout the country !

